# Pharmacokinetic–Pharmacodynamic Correlation Analysis of *Rhodiola crenulata* in Rats with Myocardial Ischemia

**DOI:** 10.3390/ph17050595

**Published:** 2024-05-07

**Authors:** Zhixin Jia, Guoming Zou, Yongyan Xie, Enning Zhang, Mureziya Yimingjiang, Xianlong Cheng, Cong Fang, Feng Wei

**Affiliations:** 1 National Institutes for Food and Drug Control, Beijing 100050, China; jessiejzx@163.com (Z.J.);; 2 Jiangxi University of Chinese Medicine, Nanchang 330004, China; zouguoming@jxutcm.edu (G.Z.); lyan8227@126.com (Y.X.); 3 School of Life Science, Beijing University of Chinese Medicine, Beijing 102401, China; 18634085688@126.com; 4 School of Chinese Materia Medical, Beijing University of Chinese Medicine, Beijing 102401, China; marziya96@163.com

**Keywords:** myocardial ischemia, pharmacokinetics, pharmacodynamics, PK-PD, *Rhodiola crenulata*

## Abstract

The pharmacokinetics (PK) of *Rhodiola crenulata* in rats were studied, and pharmacokinetic–pharmacodynamic (PK-PD) correlation analysis was performed to elucidate their time–concentration–effect relationship. The myocardial ischemia model was made with pituitrin. Rats were divided into sham operation, sham operation administration, model, and model administration groups (SG, SDG, MG, and MDG, respectively; *n* = 6). Blood was collected from the fundus venous plexus at different time points after oral administration. The HPLC-QQQ-MS/MS method was established for the quantification of five components of *Rhodiola crenulata*. CK, HBDH, SOD, LDH, and AST at different time points were detected via an automatic biochemical analyzer. DAS software was used to analyze PK parameters and PK-PD correlation. The myocardial ischemia model was established successfully. There were significant differences in the PK parameters (AUC_0–t_, AUC_0–∞_, C_max_) in MDG when compared with SDG. Two PD indicators, CK and HBDH, conforming to the sigmoid-E_max_ model, had high correlation with the five components, which indicated a delay in the pharmacological effect relative to the drug concentration in plasma. The difference in the PK parameters between modeled and normal rats was studied, and the time–concentration–effect of composition and effect indicators were investigated. This study can provide reference for the rational clinical application of Rhodiola crenulata and for related studies of other anti-myocardial ischemia drugs.

## 1. Introduction

With the improvement of modern living standards, the incidence of cardiovascular diseases has been increasing year by year. Among them, ischemic heart disease, as one of the leading causes of death worldwide, poses a serious threat to the health of humans [1,2]. Myocardial ischemia (MI) is the main pathophysiological process of ischemic heart disease, and acute or sustained MI can lead to acute myocardial infarction, accompanied by potentially lethal arrhythmias, shock, or heart failure [3]. Therefore, the research and development of anti-myocardial ischemia drugs and the elucidation of their mechanisms are key scientific issues that have attracted much attention.

*Rhodiola rosea* L. (RC), known as “golden root” or “roseroot”, belongs to the plant family Crassulaceae, and is a famous traditional Chinese medicine (TCM). In TCM applications, RC is used as a tonic, hemostatic agent, or antibacterial agent to treat and maintain cardiovascular health, as well as to combat altitude sickness and cold- and flu-like symptoms [4,5]. The utilization of RC, either as a monotherapy or in combination, is frequently employed for the management of myocardial ischemia-related disorders. The Sofren injection, which is a preparation of RC, is commonly employed in clinical settings as an intravenous Chinese medicine formulation derived from Rhodiola rosea extract, primarily utilized for the management of cardiovascular disorders [6]. The Sofren injection is specifically designed to alleviate pathological conditions in patients with cardiovascular disease by inhibiting excessive inflammatory responses, thereby improving the microenvironment [7,8]. The chemical composition of RC exhibits a complex and diverse nature, similar to other TCMs [9]. We conducted an extensive study on the medicinal constituents of RC in the initial phase, employing UHPLC-QTOF-MS coupled with a pseudotargeted analysis strategy, resulting in the identification of a total of 104 compounds. The primary constituents encompass flavanols and gallic acid derivatives, alcohols, organic acids, and their glycosides, as well as flavonoids and their glycosides. Phytochemical researches have demonstrated that salidroside and tyrosol, the main bioactive compounds, exhibit diverse pharmacological effects, including enhanced myocardial contraction and hypotensive induction [10]. Currently, there are reports available in the literature on the pharmacokinetics (PK) of salidroside in rats, with tyrosol being identified as its primary metabolite [11]. The PK characteristics and tissue distribution of salidroside in mice have also been reported [12]. Most of the relevant PK studies focused on one or several monomer compounds, and there was no correlation analysis with pharmacodynamics (PD). The PK-PD modeling analysis of the main components in rats with MI has not been reported.

PK-PD modeling is a dynamic process that fully reflects the PK process and PD indicators comprehensively in vivo, which can be used to evaluate the dynamic effects of drugs in vivo more objectively and scientifically. The application of PK-PD correlation analysis to the study of TCM theory engenders an academic system with a “holistic view”, as the essence of the complex TCM system can provide a scientific basis for elucidating the pharmacodynamic material basis and mechanisms of TCM [13,14]. In addition, PK-PD has become a major tool in clinical pharmacology, which can help optimize clinical drug delivery schedules and provide data reference for rational drug use [15].

Therefore, a combined PK-PD correlation analysis was carried out in this research to study the combined effects of the active components of RC in normal and MI rats, and to clarify the functional characteristics of these active components in rats.

## 2. Results

### 2.1. Characterization of the Myocardial Ischemia Model

**Figure 1:**
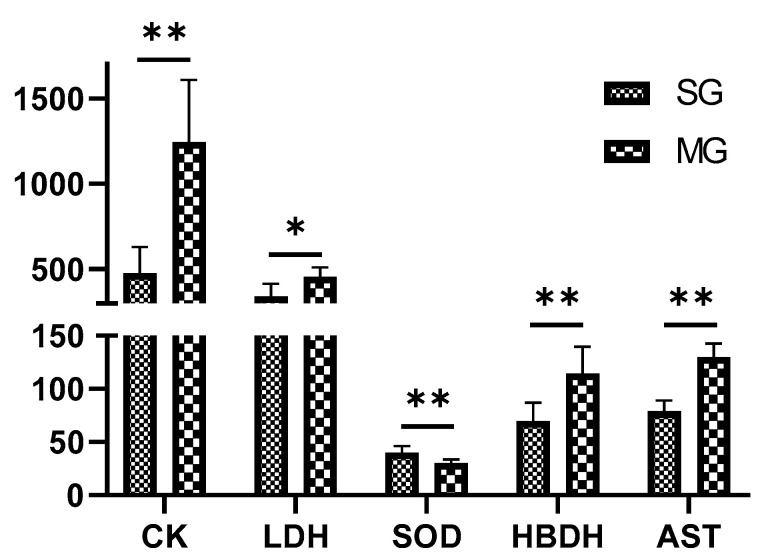
Biochemical index in the SG and MG (x ± SD, *n* = 6). ** *p* < 0.01; * *p* < 0.05.

Compared with the SG, the activities of CK, LDH, AST, and HBDH in the plasma of rats in the MG were significantly increased (*p* < 0.05, 0.01), while the activity of SOD was significantly decreased (*p* < 0.01). These results are shown in Figure 1.

**Figure 2:**
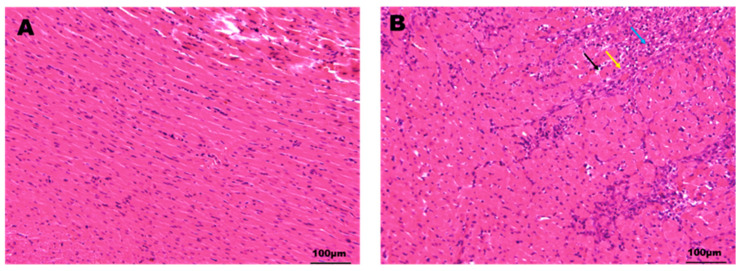
Pathological changes in heart HE staining in rats ((**A**) SG; (**B**) MG).

HE staining (Figure 2) revealed that the heart in the SG exhibited a well-defined myocardial cell structure, regular tissue morphology, a normal arrangement of myocardial fibers, and the absence of inflammatory infiltration or necrosis. In contrast, the heart in the MG displayed myocardial fiber necrosis (indicated by the black arrow), hyperchromasia or nuclear dissolution, connective tissue hyperplasia (indicated by the yellow arrow), and lymphocytic infiltration (indicated by the blue arrow). These findings strongly support the successful establishment of a pituitrin (Pit)-induced MI model in SD rats.

### 2.2. Method Validation

#### 2.2.1. Linearity, LOD, and LOQ

The calibration curves and correlation coefficients (R > 0.99) of all standard substances are shown in Table 1. The concentration range of these five compounds was between 5 and 500 ng/mL. The LOD and LOQ, which were defined as the lowest concentrations, afforded S/N ratios of 3 and 10, respectively. The LOQ values for these five detected components were 0.50–2.0 ng/mL, while the LOD values were 0.20–1.0 ng/mL. These results show that our established method is sensitive enough for plasma sample analysis.

#### 2.2.2. Specificity

**Figure 3:**
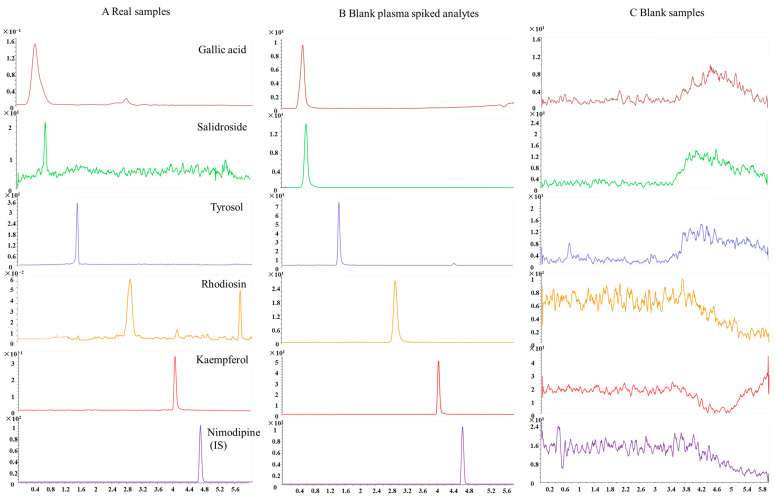
Representative MRM chromatograms for gallic acid, salidroside, tyrosol, rhodiosin, kaempferol, and IS in rat plasma. (**A**) Rat plasma samples after oral administration of RC for 30 min; (**B**) blank rat plasma spiked with TSG, EG, PG, AE, EM, and IS; (**C**) blank rat plasma.

A typical chromatogram of a plasma sample from an SD rat is shown in Figure 3. The endogenous components did not interfere with the peaks of gallic acid, salidroside, tyrosol, rhodiosin, kaempferol, and IS when compared with the chromatogram of blank rat plasma. The method has obvious specificity.

#### 2.2.3. Precision and Accuracy

The intra- and inter-day precision and accuracies of the method were summarized in Table 2. The relative standard deviation percentage (RSD%) of gallic acid, salidroside, tyrosol, rhodiosin, and kaempferol at the three concentrations were all less than 14.6%. In addition, the accuracy of the method ranged from 85.71% to 102.5%.

#### 2.2.4. Extraction Recovery and Matrix Effect

The recovery rates and matrix effects are shown in Table 3. The recoveries of gallic acid, salidroside, tyrosol, rhodiosin, and kaempferol ranged from 85.32% to 101.4%, and the RSD was less than 13.5%. The matrix effect ranges from 89.92% to 107.5%, and the RSD was less than 14.1%, which indicates that there is no obvious inhibition or enhancement of the ionization of these analytes.

#### 2.2.5. Stability

The short-term stability and long-term stability results are shown in Table 4. In all stability tests, the RSD of the test responses were within 13.6%, and the matrix effects ranged from 85.44% to 105.6%. The results showed that gallic acid, salidroside, tyrosol, rhodiosin, and kaempferol were all stable under the conditions encountered during simulated sample storage, handling, and analysis (48 h at 4 °C and 10 days in 3 freeze–thaw cycles). We observed no significant degradation, which indicated that the plasma samples were stable when treated under all test conditions.

### 2.3. PK Profiles in Normal and Myocardial-Ischemia-Modeled Rats

**Figure 4:**
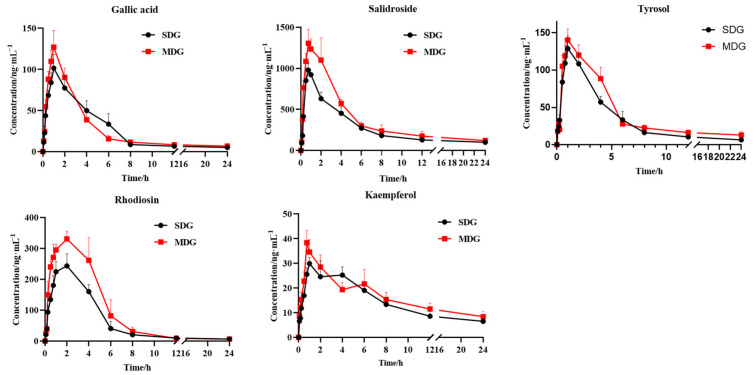
Plasma concentration–time profiles of five components in SDG and MDG rats (*n* = 6).

The drug concentration in plasma was calculated using the standard curve, and the mean blood concentration was plotted against the time to obtain the mean blood concentration–time curve (Figure 4). The PK parameters were analyzed using DAS software (3.2.8), as shown in Table 5.

It was found that, when compared with the normal rats, the metabolism of compounds in MI rats was different. In the case of salidroside, AUC_0−t_ and C_max_ increased significantly in MDG when compared with that in the SDG. For kaempferol, when compared with SDG rats, AUC_0−∞_ and C_max_ increased significantly in MDG rats. For tyrosol, AUC_0−t_ and AUC_0−∞_ increased significantly, and, in the case of rhodiosin, AUC_0−t_, AUC_0−∞_, and C_max_ increased significantly. For gallic acid, C_max_ increased significantly. We also observed a marked decreasing tendency in CL_Z_/F for tyrosol, rhodiosin, and kaempferol.

### 2.4. PD Study and PK-PD Correlation Analysis

**Figure 5:**
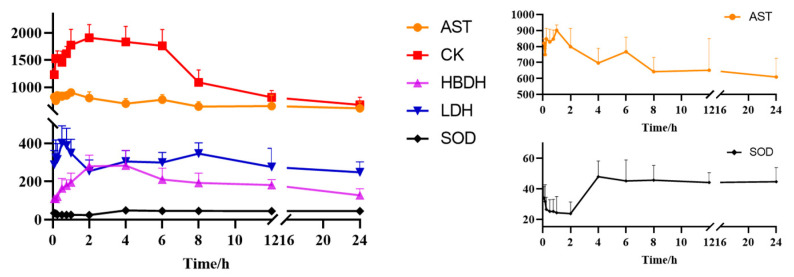
Blood concentration–time profiles of AST, CK, HBDH, LDH, and SOD (mean ± SD).

The contents of the CK, HBDH, LDH, and SOD activity of the plasma samples in the MDG at different time points were measured via an automatic biochemical analyzer and plotted according to time, as shown in Figure 5. It was found that the overall levels of AST, CK, and HBDH increased first and then decreased with time; SOD increased overall; and LDH shows a fluctuating trend.

Subsequently, we incorporated the PK and PD data to conduct PK-PD modeling through DAS. The AIC values of several PK components in vivo were 146.4, 126.2, 108.7, 102.4, and 71.9, corresponding to salidroside, rhodiosin, tyrosol, gallic acid, kaempferol, respectively, which indicates that the relationship between the plasma concentrations of these five compounds and PD levels could best be described via a sigmoid-Emax model. PK and PD were fitted, respectively, and the equation obtained was shown in Table 6. After fitting, the parameters corresponding to the PD indexes of each compound are shown in Table 7.

According to the screening criterion of *p* < 0.05, the above results indicate no significant differences between AST, LDH, and SOD indicators, thus making them unsuitable as PD markers. However, both CK and HBDH indicators have *p* values less than 0.05 and R^2^ values greater than 0.8, suggesting their suitability as PD markers for correlation analysis with these five components in vivo.

## 3. Discussion

In this study, an HPLC-QQQ-MS/MS method was used to simultaneously determine the salidroside, rhodiosin, tyrosol, gallic acid, and kaempferol contents in normal and MI rats. This established method has been well validated through linearity, specificity, recovery, precision, and stability. The established method could meet the needs of the determining of plasma samples and could be used for PK studies.

RC has been used to maintain healthy cardiovascular function, as well as being used as a candidate for the treatment of cardiovascular disease [4,5]. In early research, scholars conducted a detailed study on the plasma components of RC after oral administration, and 24 kinds of compounds were identified in vivo [16]. Phytochemical studies have revealed that salidroside and tyrosol, which were the main bioactive compounds, are similar to those used in modern medicine to improve cardiovascular function. These compounds exert pharmacological effects, such as increasing myocardial contraction, enhancing myocardial contractility, and inducing hypotension in laboratory animals [17]. Although its composition in vivo is basically clear, which compounds are pharmacodynamic substances, how they are related to PK, and what the mechanism of action is still need to be further studied.

PK and PD are important interrelated pharmacological processes, which could clarify the activity of drugs in vivo and provide references for their rational clinical application [18]. PK analysis provides insight into the relationship between the drug dose and time in the body, while PD analysis assesses the relationship between drug dose and efficacy. An accurate study of the PK-PD relationship is critical to understanding which bioactive ingredients in Chinese medicines are associated with efficacy [19]. Therefore, PK-PD correlation analysis was used in this study, linking dynamic concentration–time processes and effect–time profile relationships [20,21,22]. An analysis based on PK-PD association can provide a basis for elucidating the mechanism of action, and it can also provide a reference for the discovery of quality markers of Chinese medicine, as well as rational clinical applications. The model adopted in this study is the S-E_max_ model. In the equation, γ means the shape factor in the formula, affecting the slope and determining the steepness of the curve. E_max_ can provide the maximum response intensity, and ED_50_ is for evaluating the safety of the drug, which is understood as the amount of the drug that causes 50% of the maximum response intensity. The γ is less than 1, indicating that the flat concentration–effect curve is relative, and the efficacy varies moderately over a wide range of concentrations [23,24].

Pit can induce a coronary artery spasm and then cause MI. The rat model of acute MI prepared with Pit is similar to the pathogenesis of clinical-variant angina pectoris, and has been widely used for screening anti-myocardial ischemia drugs and in the study of related mechanisms of action [25,26,27]. MI leads to myocardial cell damage and changes in membrane permeability, resulting in a large number of enzymes, proteins, and their decomposition products being released into the blood, among which CK, LDH, AST, and HBDH are important markers by which to measure myocardial damage [28,29,30]. SOD is an active oxygen-scavenging agent that can convert superoxide anions into hydrogen peroxide in the body and then convert it into water through the action of other enzymes, which can reflect the degree of oxidative stress in the body [31]. As a preliminary PD indicator, it can be explored and studied to reasonably reflect the disease and drug mechanisms.

According to the PK results, the T_max_ of gallic acid, salidroside, and tyrosol is about 1 h, while the T_max_ of rhodiosin is about 2 h. The T_max_ of all kaempferol changed considerably after molding from 1.67 h to 0.75 h. The T_max_ of all these compounds changed, except for gallic acid, but with no significant difference, which may be related to individual differences in animals and other factors. The AUC_0−t_ and AUC_0−∞_ of each compound in MDG increased significantly, which may be related to the difference in drug absorption and the metabolic rate in a pathological state when compared with a normal state. The C_max_ of four compounds (gallic acid, salidroside, rhodiosin, and kaempferol) was significantly increased, indicating that the maximum blood concentration of these compounds was significantly increased under the disease state. The CL_Z_/F of tyrosol, rhodiosin, and kaempferol decreased significantly, indicating that the elimination rate in the disease state was slower than that in the normal state. The above results showed that, in the state of MI, these components remained in the rats’ bodies for a long time, the exposure was higher, and the clearance was lower in general. Studies have shown that RC may synergistically inhibit myocardial apoptosis and improve MI-reperfusion injury through the anti-inflammatory regulation of energy metabolism and oxidative stress, which may be related to the HIF-1/VEGF/PI3K-Akt signaling pathway [32]. Further research found that the systemic exposure to salidroside in hypoxia increased significantly, which may be related to the weakening of liver metabolism under the disease state [33]. This conclusion is consistent with our results. It has also been reported that the bioavailability of salidroside and its metabolite tyrosol increased significantly under pathological conditions. This may be due to the upregulation of the transporter protein (SGLT1) in the disease state, the increased permeability of intestinal epithelial cells and vascular endothelial cells (through destroying brush border microvilli and decreasing tight junction protein ZO-1 expression), and then the increase in blood drug concentration [34,35]. Kaempferol could inhibit P-GP-mediated effluents in the small intestine or CYP3A4-mediated metabolism in the small intestine or liver or both, thereby affecting the body’s absorption of other components [36]. In addition, kaempferol increased AUC, MRT, t_1/2_, and C_max_ significantly, and decreased clearance in the modeled rats, which may be related to the significantly decreased mRNA and protein expression of CYP1A2 and UGT1A9 in the disease state [37]. The PK characteristics of gallic acid in the modeled rats with myocardial infarction induced by isoproterenol were significantly different from those of normal rats, with prolonged T_1/2_ and MRT and significantly decreased CL_Z_/F, which may be related to changes in intestinal epithelial membrane permeability [38].

After model fitting, AST, LDH, and SOD, which were initially selected as PD indicators in this study, showed no significant differences. Therefore, they were deemed unsuitable for correlation analysis with these five components. Conversely, CK and HBDH exhibited *p* values less than 0.05 and were considered suitable. AST and LDH can reflect not only heart injury, but also liver injury; hence, their low specificity may explain their unsuitability as PD indicators in this study. CK is a classical myocardial enzyme detection index that sensitively reflects the degree of myocardial damage with rapid changes [28]. HBDH can indicate red blood cell damage and exhibits higher sensitivity during acute MI [29]. These reasons may elucidate the strong correlation between these two biochemical indicators and the five body components observed in this study. When compared with the T_max_ of the five components, the change in drug effect intensity showed an obvious lag effect. According to the literature, the existence of hysteresis has been found in studies of the PK-PD combination of many active ingredients in Chinese medicine and chemical drugs [39,40]. The results showed that only the relationship between concentration and time was unable to reflect the whole process of the drug effect in vivo. Only the relationship between concentration, time, and effect could guide clinical rational drug use more effectively. The hysteresis phenomenon in this study may be due to the gradual distribution of drugs into cardiac muscle, vascular smooth muscle, and other effect sites, alongside the slow uptake of drugs by relevant cells from tissue fluid or blood, resulting in the change in their concentration lagging behind the change in blood concentration, as well as the resulting pharmacological effect lagging behind the blood concentration.

TCM has a long history of use and excellent clinical effects, but its mechanism of action is still little known and needs to be further clarified. TCM usually consists of multicomponent therapies, which are used to treat system-wide disorders. Thus, TCM was commonly considered to be a “system-to-system” approach [41]. However, due to the complexity of the composition, action mechanism, and material basis of TCMs, related research is extremely difficult [42]. The PK-PD model is a powerful tool for studying the connection between the dynamic change of drug concentration and the variation in drug efficacy comprehensively, and it has an important role to play in elucidating the bases and mechanism of pharmacodynamic substances. Furthermore, except for various kinds of concentration–effect relationships, PK-PD models could also describe complicated disease dynamics or response effects to specific drug doses over time [43].

## 4. Materials and Methods

### 4.1. Chemicals and Reagents

**Figure 6:**
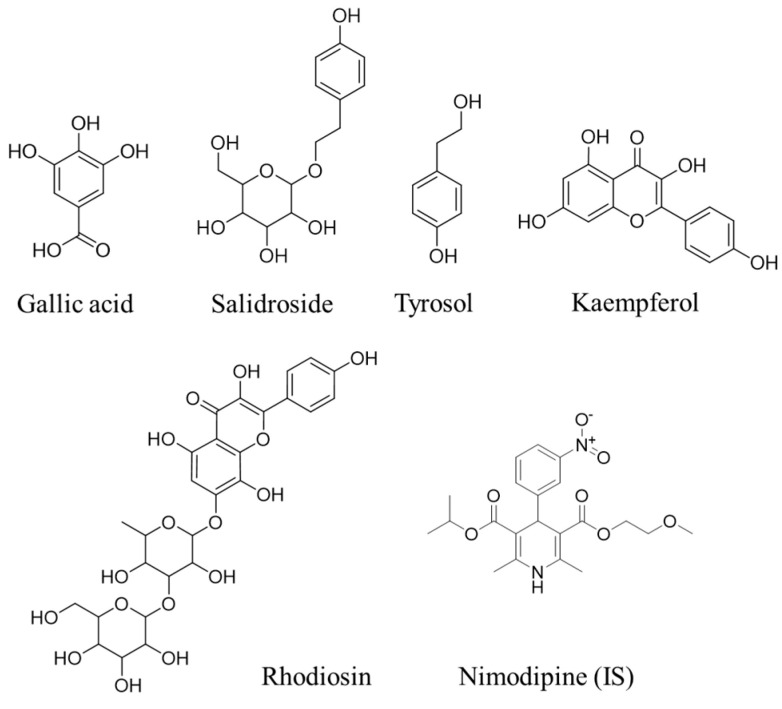
Chemical structures of gallic acid, salidroside, tyrosol, rhodiosin, kaempferol, and nimodipine (IS).

Standards including gallic acid (batch No. RFS-M01711812016, purity ≥ 98%), salidroside (batch No. H-040-180727, purity ≥ 98%), tyrosol (batch No. L-042-170426, purity ≥ 98%), rhodiosin (batch No. H-070-171211, purity ≥ 98%), and kaempferol (batch No. RFS-S01411809008, purity ≥ 98%) were purchased from Chengdu Ruifensi Biotechnology Co., Ltd., Chengdu, China. Nimodipine (internal standard (IS), batch No. 912A027, purity ≥ 98%) and heparin sodium (batch No. 1231Q025, size ≥ 140 units/mg) were purchased from Beijing Solaibao Technology Co., Ltd., Beijing, China. Pit (batch No. 20210811) was purchased from Zhongda Veterinary Medicine Co., Ltd., Harbin, China. The structural formula of standards and IS is shown in Figure 6.

Methanol and acetonitrile (LC-MS grade) were obtained from Fisher company. Analytical grade formic acid and ethyl alcohol were obtained from Sigma-Aldrich (St. Louis, MO, USA), and water (ultra-pure) was purified by a Milli-Q Ultrapure water system from Merck Millipore (Bedford, MA, USA).

### 4.2. Instruments and Chromatographic Conditions

The assay was conducted using a 1260 Agilent high-performance liquid chromatography system, coupled with a 6470Agilent QQQ MS (Agilent Technologies, Santa Clara, CA, USA). Chromatography was performed on an Agilent ZORBAX Eclipse Plus C18 column (50 mm × 2.1 mm, 1.8 μm) at a temperature of 40 °C. The mobile phase consisted of 0.1% formic acid water (A) -acetonitrile (B), and the flow rate was set at 0.4 mL/min. Gradient elution was carried out as follows: from 0 to 1.5 min, the mobile phase contained 20% B; from 3 to 4 min, it transitioned from 80% to 100% B; at the eighth minute, it reached a composition of pure B. A post time of five minutes followed, and the injection volume was set at 5 μL. The quantitative analysis employed multiple reaction monitoring (MRM) in a negative ion mode using Dual AJS ESI source conditions, including gas temperature set at 175 °C; gas flow maintained at 14 L/min; nebulizer pressure adjusted to 40 psi; sheath gas temperature kept constant at 300 °C; sheath gas flow regulated to 12 L/min; capillary voltage set at500 V (−); nozzle voltage fixed at 1000 V; and fragmentor voltage established as 380 V. The precursor ions and product ions for the detected compounds are listed in Table 8.

### 4.3. The Preparation of RC

A total of 500 g of rhodiola rosea decoction pieces were weighed and placed in a 10 L round-bottom flask, followed by the addition of 4 L of 95% ethanol. The mixture was heated and refluxed for 1.5 h, subjected to two extractions, and the extracted liquid was then steam distilled using a rotary evaporator until it reached near dryness. Subsequently, the resulting residue was transferred onto a stainless-steel plate and vacuum dried at 60 °C until it transformed into powder form, yielding a final extract weight of 131.7 g.

### 4.4. Experimental Animals and Sample Collection

Male SD rats (weight 200 ± 5 g) were purchased from SPF (Beijing) Biotechnology Co., Ltd., Beijing, China, and all the animals were maintained under the above conditions for 7 days before the start of the experiment for acclimatization. The rats were kept with ad libitum access to water and standard food, with a natural light–dark cycle (25 ± 2 °C and 40–50% humidity). All the animal procedures were in accordance with the Regulations of Experimental Animal Administration, issued by the State Committee of Science and Technology of the People’s Republic of China. The animal ethics were approved by the Animal Care and Use Committee of the Beijing University of Chinese Medicine (BUCM) with the approval number of BUCM-4-2021083102-3044.

The rat model of MI was made with Pit. The rats were divided into a sham operation group (SG), sham operation administration group (SDG), model group (MG), and model administration group (MDG) (*n* = 6 for each group). The MI model was prepared via an intraperitoneal injection (a single time) of Pit in the MG and MDG, with a dosage of 20 U/kg and a volume of 10 mL/kg. At the same time, the rats in the SG and SDG groups were given the same volume of saline in the same way.

RC extract (2 g/kg, equivalent to human clinical equivalent dose) was given to rats via oral administration in SDG and MDG, and the same volume of normal saline was orally administered to the SG and MG. Rats were fasted for 12 h before the experiment. Blood was collected from the fundus venous plexus at 0, 5, 10, 15, 30, 45 min, 1, 2, 4, 6, 8, 12, and 24 h after in gavage, placed in a centrifuge tube impregnated with heparin sodium, centrifuged at 3500 rpm for 15 min, and then the plasma was collected and divided into two parts. One part was used for biochemical index determination and the other part was used to determine the content of pharmaceutical ingredients in vivo. After the experiment, the rats were sacrificed, and the hearts were placed in paraformaldehyde for histopathological analysis.

### 4.5. Biochemical Index Detection and Histopathology

Plasma creatine kinase (CK), hydroxybutyrate dehydrogenase (HBDH), lactate dehydrogenase (LDH), and superoxide dismutase (SOD) activity was determined using an automatic biochemical analyzer.

Hematoxylin–eosin (HE) staining was used to stain the target organs in order to observe the effects of drugs on rat hearts before and after modeling. The heart fixed with paraformaldehyde was dehydrated, embedded, sliced, and stained; then, it was observed under an optical microscope, and the main parts were photographed.

### 4.6. The Preparation of Plasma Samples

The plasma sample of 100 μL was accurately absorbed, 50 μL internal standard solution (Nimodipine, 500 ng/mL) was added, then 300 μL acetonitrile precipitated protein was added, vortex for 10 min, placed under ultrasound for 3 min, centrifuged at 13,000 rpm for 10 min, and then the supernatant was transferred to another centrifuge tube. It was then concentrated until dry with a rotary evaporator. The residue was redissolved with 100 μL 50% acetonitrile, swirled for 1 min, placed under ultrasonic for 3 min, centrifuged at 13,000 rpm for 10 min, and the supernatant was taken for analysis.

### 4.7. Methodological Validation

A mixed reference reserve solution of gallic acid, salidroside, tyrosol, rhodiosin, and kaempferol was prepared at 500 ng/mL, respectively, and diluted to 200, 100, 50, 20, 10, and 5 ng/mL in sequence. A series concentration mixed reference solution with different concentrations was added to 100 μL plasma, and was treated according to the method under Section 4.6 to obtain the matrix standard curve sample. Samples with concentrations of 400, 100, and 10 ng/mL, respectively, were taken as quality control (QC) samples of high, medium, and low concentrations (HQC, MQC, LQC). The method was validated, including linearity, inter-day/intra-day precision, repeatability, stability, extraction recovery, and matrix effect, according to FDA bioanalytical method validation guidelines.

### 4.8. PK Analysis

A validated HPLC-QQQ-MS/MS method was used for the determination of drug-derived components in plasma samples at different time points. The accompanying standard curve was substituted to calculate the content of the compounds to be measured, and then the PK parameters were calculated using DAS software (version 3.2.8), and the blood concentration–time curve was plotted using GraphPad Prism (version 8.0.2). PK behavior was compared between the sham operation and model group, and PK models were established, respectively.

### 4.9. PD Study and PK-PD Analysis

The activity of the CK, HBDH, LDH, and SOD of plasma samples collected at different time points was determined using an automatic biochemical analyzer for PD index evaluation. The PK parameters for five components (gallic acid, salidroside, tyrosol, rhodiosin, and kaempferol) were analyzed. Two compartmental methods were used, employing the drug and statistics, which was supplied by the Chinese Pharmacological Society. Then, the efficacy indicators were fitted in the PK-PD model.

### 4.10. Statistical Analysis

Statistical analysis was carried out using GraphPad software (version 8.0.2). A two-tailed *p*-value with the criterion of less than 0.05 was regarded as significant. Mean ± standard deviation (SD) was used to express the data.

## 5. Conclusions

In this study, a HPLC-QQQ-MS/MS method was successfully established and employed to determine the plasma concentration of five components (gallic acid, salidroside, tyrosol, rhodiosin, and kaempferol) in rats with myocardial ischemia and normal rats. Five preliminary pharmacodynamic indicators (AST, CK, HBDH, LDH, and SOD) were selected and combined with RC compositions in vivo to establish PK-PD combination models for MI-modeled rats. Two suitable PD indicators were further screened for analysis. In the PK analysis, significant changes were observed in the PK parameters of the five components in vivo as follows: AUC_0−T_ and AUC_0−∞_ increased significantly; C_max_ also increased significantly; and CL_Z_/F decreased significantly. These results indicated higher drug exposure levels in vivo for modeled rats when compared to normal ones. In the PD study, the levels of AST, CK, and HBDH first increased and then decreased over time; SOD showed an overall increasing trend; and LDH fluctuated. The S-E_max_ model was used, and the *p* value for CK and HBDH were both below 0.05, with the R^2^ above 0.8, which indicated that these two PD indicators were suitable for correlation analysis with the five components absorbed in rats. This comprehensive study investigated the correlations between the drug dosage, the duration of action in vivo, as well as the drug dosage efficacy relationship, providing valuable data support towards elucidating the mechanism of action of RC treatment of MI, while also offering a reliable reference for its rational clinical application and further development.

## Figures and Tables

**Table 1:** Standard curves, correlation coefficient (R), LOQ, and LOD.

Name	Curve	Correlation Coefficient (R)	LOQ (ng/mL)	LOD (ng/mL)
Gallic Acid	y = 0.3595x + 1.098	0.9996	0.5	0.2
Salidroside	y = 0.0441x + 0.6128	0.9978	1	0.5
Tyrosol	y = 0.0605x + 0.5116	0.9991	2	1
Rhodiosin	y = 0.0708x − 0.1770	0.9992	2	1
Kaempferol	y = 0.0532x − 0.1185	0.9998	1	0.5

**Table 2:** Precision and accuracy of the method for the determination of gallic acid, salidroside, tyrosol, rhodiosin, and kaempferol in rat plasma (*n* = 6).

Analytes	Spiked Conc. (ng/mL)	Intra-Day		Inter-Day	
		RSD (%)	RE (%)	RSD (%)	RE (%)
Gallic Acid	10	11.9	93.13	12.7	89.24
	50	5.77	96.28	8.26	95.91
	400	5.62	99.88	3.11	97.25
Salidroside	10	5.32	95.61	8.32	88.94
	50	4.71	99.72	3.55	100.1
	400	6.89	101.3	5.28	98.12
Tyrosol	10	9.74	96.54	14.7	86.31
	50	8.09	91.09	9.55	91.83
	400	4.32	92.38	8.28	95.62
Rhodiosin	10	13.2	85.71	14.1	86.22
	50	9.85	89.92	6.12	90.14
	400	4.30	93.24	6.71	92.53
Kaempferol	10	3.77	101.7	9.08	93.27
	50	7.91	99.84	2.13	102.5
	400	6.32	98.52	5.56	97.95

**Table 3:** Recovery and matrix effect of gallic acid, salidroside, tyrosol, rhodiosin, and kaempferol (*n* = 6).

Analytes	Spiked Conc. (ng/mL)	Extraction Recovery		Matrix Effect	
		RSD (%)	RE (%)	RSD (%)	RE (%)
Gallic Acid	10	12.7	88.62	14.1	89.92
	50	6.58	95.73	8.40	92.33
	400	8.31	100.1	5.64	99.87
Salidroside	10	5.32	90.53	6.90	104.2
	50	6.43	92.74	3.38	102.3
	400	3.91	95.71	4.19	99.11
Tyrosol	10	13.5	85.32	12.6	92.22
	50	9.82	87.63	3.55	107.5
	400	8.28	89.27	6.39	98.30
Rhodiosin	10	10.1	87.01	7.47	103.3
	50	5.32	92.39	7.51	95.78
	400	6.29	90.01	3.22	97.85
Kaempferol	10	5.11	95.52	5.36	99.01
	50	4.37	99.03	2.77	103.7
	400	3.18	101.4	4.12	93.61

**Table 4:** Stability results of gallic acid, salidroside, tyrosol, rhodiosin, and kaempferol in rat plasma under different conditions (*n* = 6).

Analytes	Spiked Conc. (ng/mL)	4 °C, 48 h		−80 °C, 10 Days		Three Freeze–Thaw Cycles	
		RSD (%)	RE (%)	RSD (%)	RE (%)	RSD (%)	RE (%)
Gallic Acid	10	3.65	100.2	9.73	99.77	10.1	98.58
	50	8.41	103.3	6.72	98.31	3.92	105.6
	400	7.22	99.11	7.69	96.65	4.44	90.26
Salidroside	10	6.37	89.72	10.2	88.35	13.6	90.22
	50	7.58	90.35	5.39	100.1	7.58	101.3
	400	4.39	98.56	6.23	97.63	8.31	99.82
Tyrosol	10	12.1	85.44	13.0	99.52	12.7	94.31
	50	9.56	90.28	9.32	93.68	7.32	96.93
	400	4.33	93.15	6.18	89.70	5.26	97.75
Rhodiosin	10	5.42	103.8	6.27	92.37	8.24	88.83
	50	7.26	95.62	6.11	100.9	3.71	98.28
	400	5.30	93.17	4.33	90.82	5.92	93.72
Kaempferol	10	4.63	101.5	5.51	97.80	4.39	102.9
	50	6.01	90.22	6.29	99.12	6.67	93.25
	400	3.99	92.55	3.72	100.4	5.10	94.57

**Table 5:** PK parameters of five compounds in SDG and MDG rat groups.

PK Parameters	Gallic Acid		Salidroside		Tyrosol		Rhodiosin		Kaempferol	
	SDG	MDG	SDG	MDG	SDG	MDG	SDG	MDG	SDG	MDG
AUC_(0−t)_ (μg·h/L)	474.84 ± 112.26	530.79 ± 60.89	5714.19 ± 320.74	7789.88 ± 1156.98 **	653.07 ± 63.25	837.42 ± 66.81 **	1190.07 ± 56.37	1736.61 ± 257.41 **	306.11 ± 30.50	354.36 ± 50.37
AUC_(0−∞)_ (μg·h/L)	549.85 ± 168.90	664.23 ± 220.79	8347.26 ± 2313.72	10,049.064 ± 1918.20	728.65 ± 49.16	1102.46 ± 257.50 **	1208.73 ± 44.35	1754.66 ± 238.70 **	427.39 ± 95.37	645.08 ± 178.86 *
MRT_(0−)_ (h)	4.32 ± 1.26	5.53 ± 0.59	7.14 ± 0.51	6.70 ± 0.61	5.59 ± 0.50	6.54 ± 0.91 *	4.28 ± 0.27	3.94 ± 0.34	8.54 ± 0.55	9.05 ± 0.70
MRT_(0−∞)_ (h)	8.92 ± 5.98	13.64 ± 10.57	13.64 ± 10.57	16.71 ± 9.83	9.86 ± 5.68	16.81 ± 9.96	4.75 ± 1.08	4.3 ± 0.40	19.41 ± 9.37	30.85 ± 14.10
*t*_1/2z_ (h)	10.25 ± 8.73	13.76 ± 10.86	19.89 ± 16.03	15.44 ± 11.21	10.02 ± 7.64	15.56 ± 0.79	4.18 ± 2.26	3.65 ± 2.87	14.11 ± 8.42	22.74 ± 10.65
T_max_ (h)	0.92 ± 0.13	0.92 ± 0.13	0.75 ± 0.16	0.83 ± 0.13	1.17 ± 0.41	0.96 ± 0.10	1.83 ± 0.41	2.5 ± 1.23	1.67 ± 1.21	0.75 ± 0
V_z_/F (L/kg)	116.47 ± 83.12	134.30 ± 68.90	15.21 ± 8.77	10.70 ± 7.46	97.04 ± 70.23	94.38 ± 44.37	25.06 ± 25.06	16.21 ± 14.31	224.28 ± 107.33	245.31 ± 67.03
CL_z_/F (L/h/kg)	9.88 ± 3.09	8.13 ± 2.21	0.64 ± 0.18	0.52 ± 0.12	6.89 ± 0.45	4.75 ± 1.12 **	4.14 ± 0.15	2.89 ± 0.39 **	12.17 ± 2.57	8.42 ± 2.93 *
C_max_ (μg/L)	102.79 ± 16.22	129.74 ± 19.93 *	1010 ± 84.17	1348.87 ± 133.39 **	132.17 ± 12.64	143.38 ± 1.41	259.08 ± 30.04	336.63 ± 22.81 **	31.23 ± 1.53	38.40 ± 4.92 **

Mean ± SD (*n* = 6) compared with SDG rats. ** *p* < 0.01; * *p* < 0.05.

**Table 6:** PK-PD model equation (*n* = 6).

	Salidroside	Rhodiosin	Tyrosol	Gallic Acid	Kaempferol
AST	E = 1091.001C^0.233^/(0.01^0.233^ + C^0.233^)	E = 1084.02C^0.397^/(0.011^0.397^ + C^0.397^)	E = 1213.835C^0.38^/(0.01^0.38^ + C^0.38^)	E = 1375.057C^0.208^/(0.01^0.208^ + C^0.208^)	E = 1422.742C^0.451^/(0.01^0.451^ + C^0.451^)
CK	E = 2288.494C^0.717^/(0.14^0.717^ + C^0.717^)	E = 2289.293C^0.814^/(0.04^0.814^ + C^0.814^)	E = 2289.882C^0.904^/(0.017^0.904^ + C^0.904^)	E = 2290.745C^0.614^/(0.009^0.614^ + C^0.614^)	E = 2736.472C^0.997^/(0.01^0.997^ + C^0.997^)
HBDH	E = 4898.547C^0.313^/(5373.93^0.313^ + C^0.313^)	E = 4335.008C^0.318^/(779.167^0.318^ + C^0.318^)	E = 7149.416C^0.355^/(579.037^0.355^ + C^0.355^)	E = 3540.625C^0.304^/(179.07^0.304^ + C^0.304^)	E = 6565.084C^0.0.405^/(50.003^0.405^ + C^0.405^)
LDH	E = 482.587C^0.184^/(0.01^0.184^ + C^0.184^)	E = 484.31C^0.292^/(0.01^0.292^ + C^0.292^)	E = 483.057C^0.481^/(0.01^0.481^ + C^0.481^)	E = 580.413C^0.189^/(0.01^0.189^ + C^0.189^)	E = 599.812C^0.467^/(0.01^0.467^ + C^0.467^)
SOD	E = 71.79C^0^/(0.01^0^ + C^0^)	E = 72.933C^0.01^/(1.173^0.01^ + C^0.01^)	E = 68.804C^0.078^/(0.01^0.078^ + C^0.078^)	E = 73.435C^0.01^/(1.484^0.01^ + C^0.01^)	E = 68.398C^0.995^/(0.01^0.995^ + C^0.995^)

**Table 7:** PK-PD model with pharmacodynamics parameters.

		AST	CK	HBDH	LDH	SOD
salidroside	E_max_	1091.001	2288.494	4898.547	482.587	71.79
	ED_50_	0.01	0.14	5373.93	0.01	0.01
	γ	0.233	0.717	0.313	0.184	0
	Ke0	2.821	2.698	0.46	2.476	0.025
	R^2^	0.559	0.848	0.91	0.349	0
	*p*	0.153	0.007	0.002	0.429	1
rhodiosin	E_max_	1084.02	2289.293	4335.008	484.31	72.933
	ED_50_	0.011	0.04	779.167	0.01	1.173
	γ	0.397	0.814	0.318	0.292	0.01
	Ke0	2.998	2.998	0.469	2.998	0.055
	R^2^	0.614	0.857	0.901	0.401	0.001
	*p*	0.106	0.006	0.002	0.347	1
tyrosol	E_max_	1213.835	2289.882	7149.416	483.057	68.804
	ED_50_	0.01	0.017	579.037	0.01	0.01
	γ	0.38	0.904	0.355	0.481	0.078
	Ke0	2.998	2.998	0.724	2.848	0.043
	R^2^	0.614	0.861	0.941	0.384	0.005
	*p*	0.106	0.006	0	0.372	0.998
gallic acid	E_max_	1375.057	2290.745	3540.625	580.413	73.435
	ED_50_	0.01	0.009	179.07	0.01	1.484
	γ	0.208	0.614	0.304	0.189	0.01
	Ke0	2.998	1.909	0.439	2.998	0.022
	R^2^	0.656	0.834	0.903	0.407	0.009
	*p*	0.076	0.009	0.002	0.338	1
kaempferol	E_max_	1422.742	2736.472	6565.084	599.812	68.398
	ED_50_	0.01	0.01	50.003	0.01	0.01
	γ	0.451	0.997	0.405	0.467	0.995
	Ke0	2.998	2.998	0.928	2.998	0.115
	R^2^	0.417	0.816	0.902	0.333	0.415
	*p*	0.323	0.013	0.002	0.455	0.327

**Table 8:** MRM conditions for each reference standard.

Compound Name	Precursor Ion (*m*/*z*)	Product Ion (*m*/*z*)	Fragmentor (V)	Collision Energy (V)
Gallic acid	169.0	125.1	90	15
Salidroside	299.1	119.0	115	30
Tyrosol	137.0	119.1	110	20
Rhodiosin	609.3	301.2	130	35
Kaempferol	285.2	151.1	160	20
Nimodipine (IS)	417.2	122.1	100	22

## Data Availability

The data presented in this study are available on request from the corresponding author (accurately indicate status).

## Abbreviations

CK, plasma creatine kinase; MI, myocardial ischemia; HBDH, hydroxybutyrate dehydrogenase; PK, pharmacokinetic; PD, pharmacodynamic; RC, Rhodiola crenulate; SG, sham operation group; MG, model group; MDG, model administration group; TCM, traditional Chinese medicine; IS, internal standard; MRM, multiple reaction monitoring; LDH, lactate dehydrogenase; SOD, superoxide dismutase; HE, hematoxylin–eosin; QC, quality control; Pit, pituitrin.
